# Supplementary material for: Movi: A fast and cache-efficient full-text pangenome index
Source: iScience. 2024 Nov 27;27(12):111464. doi: 10.1016/j.isci.2024.111464 (PMC11696632; doi:10.1016/j.isci.2024.111464)
Supplement: Document S1. Figures S1–S4, Tables S1–S8, and Data S1–S3 [file mmc1.pdf]

iScience, Volume *27*

Supplemental information

Movi: A fast and cache-efficient full-text pangenome index

Mohsen Zakeri, Nathaniel K. Brown, Omar Y. Ahmed, Travis Gagie, and Ben Langmead

```
Data: Move table (M), current run index (i), current BWT offset (j)
Result: Index of run containing LF[j]
j′ ← M[i].π + (j − M[i].p)
i′ ← M[i].ξ
while M[i′].p + M[i′].l ≤ j′ do
    i′ ← i′ + 1;
end
return i′
```

Data 1: Related to STAR Methods. The fast forward algorithm (“ff”) used in Algorithm 2.

```
Data: Move table (M), and pattern (p), number of runs (r), length of text (n)
Result: pml: pseudo matching lengths for the pattern P
ℓ ← 0, i ← r
j ← n
for k ← p.len to 1 do
    c ← p[k]
    if c ≠ M[i].c then
        if (j − M[i].p) < M.Thresh[c] then
            i ← RepositionUp(c, i)
        else
            i ← RepositionDown(c, i)
        end
        ℓ ← 0
    end
    pml[k] ← ℓ
    i′ ← ff(M, i, j)
    j′ ← M[i].π + (j − M[i].p)
    i ← i′, j ← j′
    ℓ ← ℓ + 1
end
return pml
```

Data 2: Related to STAR Methods. PML computation using move structure. RepositionUp and RepositionDown are performed using scanning in the default mode, or the explicit pointers in the constant mode.

Data: Move table (M), and patterns (R), number of simultaneous reads to process(S)
Result: Generates all the PMLs for the patterns in R

```
Strands ← {}
for i ∈ S do
    s ← NextRead(R)
    Strands.add(s)
end
while hasReads(R) do
    for s ∈ Strands do
        NextPML(M, s)
        s.readPos ← s.readPos − 1
        if s.readPos < 0 then
            WritePMLs(s)
            s ← NextRead(R)
        else
            Prefetch(s.NextID)
        end
    end
end
```

Data 3: Related to STAR Methods. The prefetching algorithm in move structure for computing PMLs. Each read is assigned to a class called "Strand" to be processed. After all the PMLs for a read in one Strand is computed, the Strand is updated to process the next read, "NextRead" retrieves the next read from the input file and assigns it to a strand. "NextPML" generates the pseudo matching length for the next base in the read assigned to strand s. "Prefetch" triggers an asynchronous retrieaval of the memory containing the destination row of the LF-mapping, which will be used later.

# of Salmonella genomes	Length (n)	# of k-mers	# of BWT runs (r)	n/r
1	9,902,766	4,881,178	6,978,104	1.42
310	2,989,425,474	37,715,191	43,006,823	69.52
620	6,015,699,904	63,682,415	71,726,597	83.86
930	9,066,651,194	76,936,737	85,953,101	105.52
1,240	12,135,301,640	85,000,303	94,849,015	127.95
1,550	15,176,878,991	93,402,839	104,050,226	145.84
all - 1,564	15,314,650,625	93,374,177	104,082,950	147.13

Table S 1: Related to STAR Methods. Total number of distinct k-mers and the number of BWTruns for an increasing number of Salmonella genomes. The BWTis built over the forward and reverse complement of genomes. Both the forward and reverse complement of each k-mer are represented by a single canonical k-mer (The lexicographically smaller k-mer).

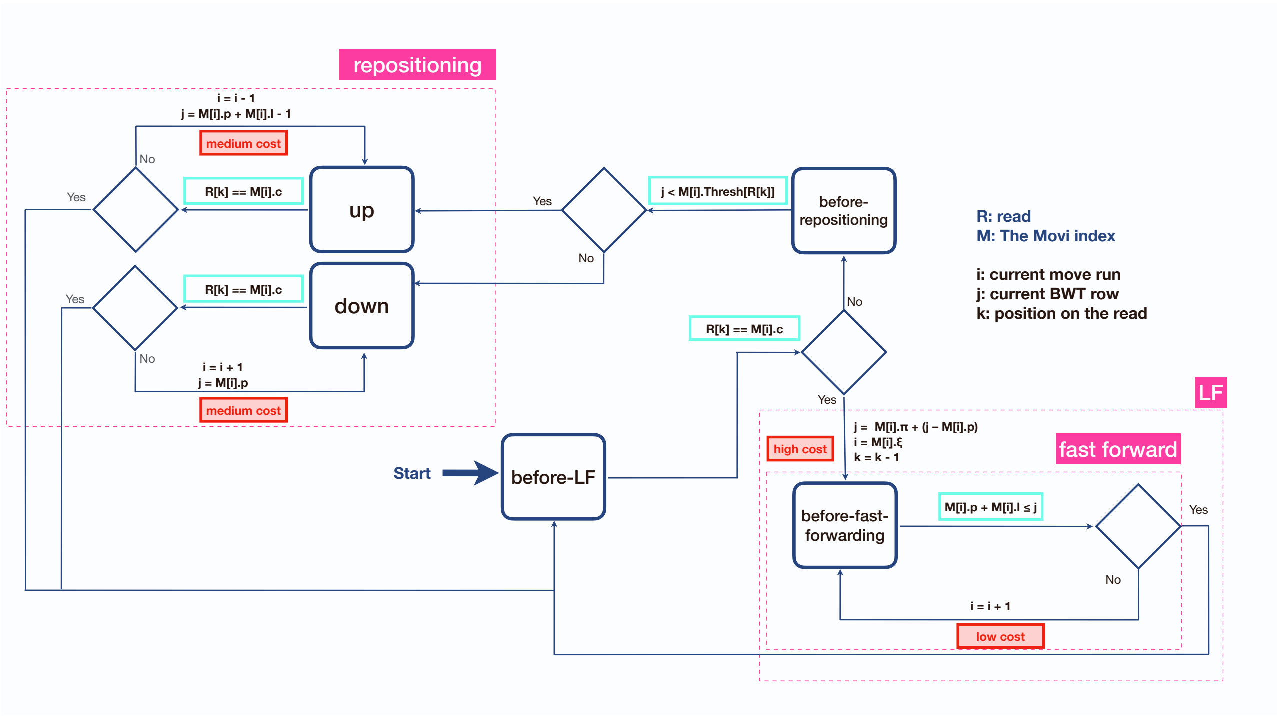

Figure S 1: Related to STAR Methods. Full schematic of the pseudo matching length (PML) computation with Movi-default. For Movi-constant pointers and direct-address lookups are used instead of the linear-scan loops shown in the figure.

Mode	prefetch	size (GB)	query (hh:mm:ss)
default	no	8.5	00:45:22
constant	no	14	00:57:28
default	yes	8.5	**00:18:40**
constant	yes	14	00:24:01

Table S 2: Related to Section 3.1. Comparison of different Movi modes. "Constant" refers to the mode that uses both (a) Nishimoto and Tabei's splitting procedure for ensuring a constant number of fast forwards and (b) constant-time direct-address lookups in the repositioning procedure. "Default" mode does not use splitting and uses linear scans in the repositioning procedure. "Prefetch" refers to whether Movi is using its latency-hiding approach. When "prefetch" is "yes," Movi is processing 16 reads concurrently and using prefetch instructions to hide cache-miss latency.

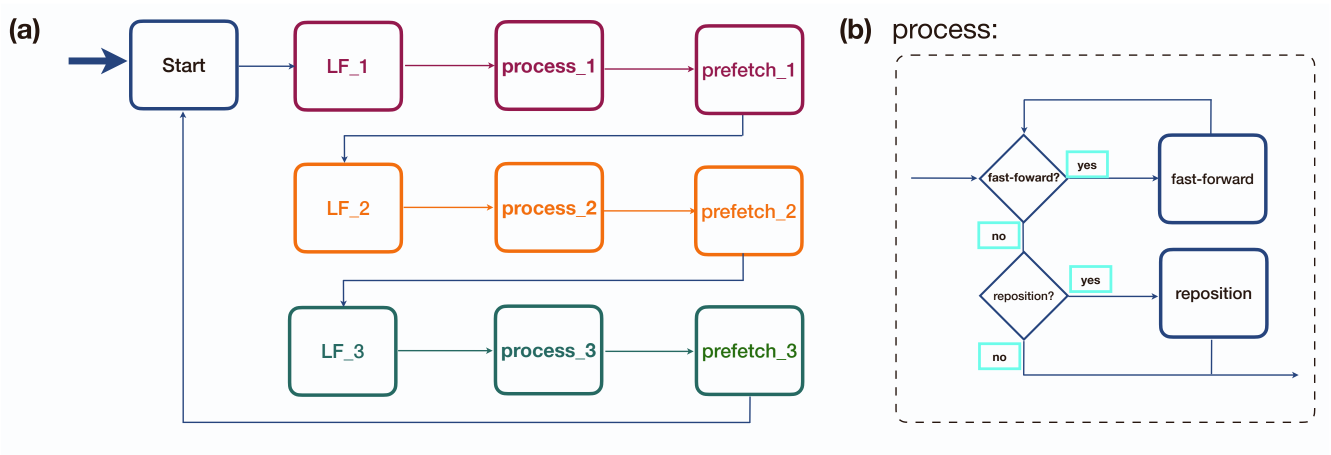

Figure S 2: Related to STAR Methods. Computing PMLs with prefetching in Movi. (a) Shows how each read is processed until the LF step which is the highest cost. Then a prefetching step is triggered to fetch the memory required to do the LF. While the memory is being prefetched, the processor moves to processing another read for which the memory required is already prefetched, (b) The process which is performed after the LF for each read, note that all the steps in this processa are low cost or medium cost, therefore, these are much faster compared to the LF step for which the memory is prefetched.

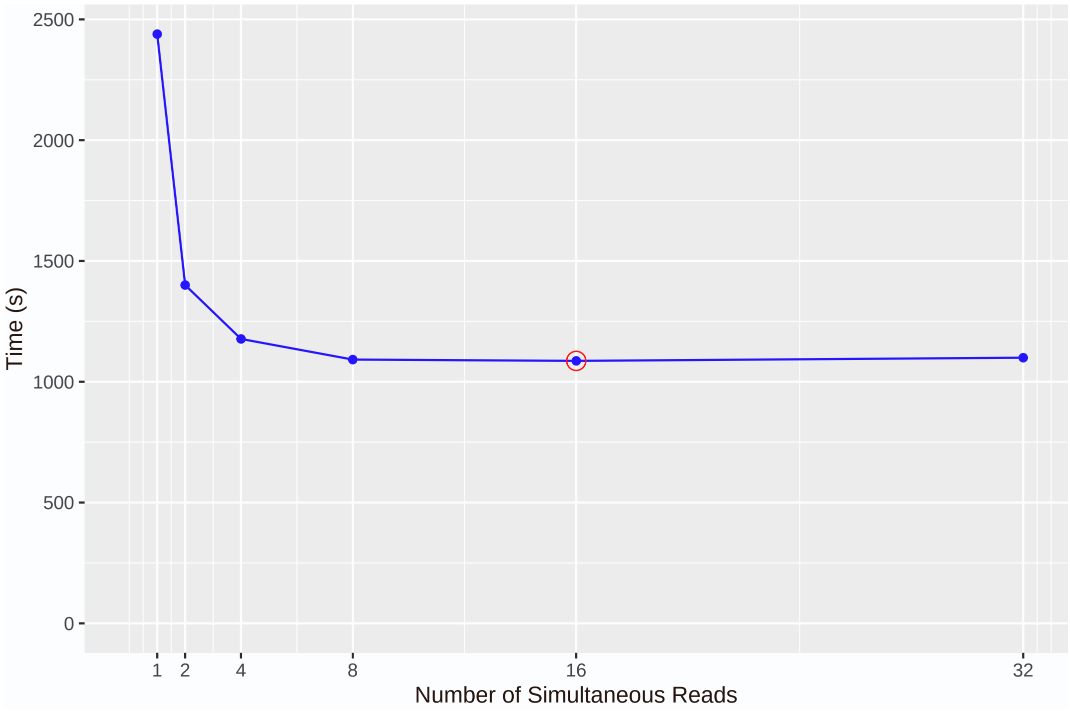

Figure S 3: Related to STAR Methods. Time required to process the reads from the Zymo community using the latency-hiding strategy as a function of the number of reads being processed concurrently. The latency-hiding benefit accrues rapidly up to 8 threads, then plateaus. Movi processes 16 concurrent reads by default (red circle). All the experiments are performed on a machine with the last-level cach (L3) size of 36 MB and a cache line of 64 Bytes.

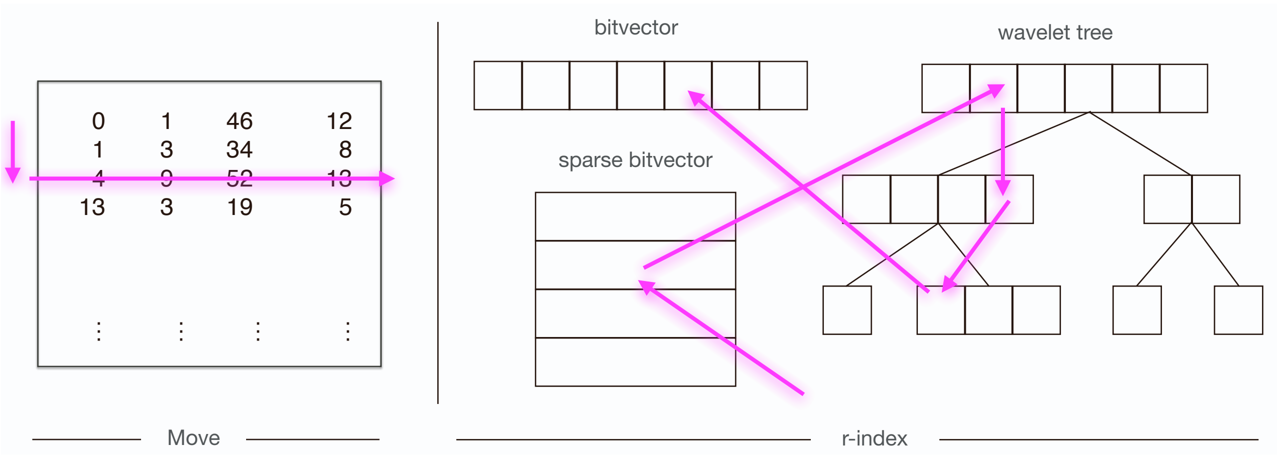

Figure S 4: Related to STAR Methods. Approximate schematic for illustrating how memory accesses are induced for `LF`-mapping in r-index and move structure. This is not an exact representation of how queries work in these indexes and is just intended to roughly show the memory access patterns.

Tool	index (GB)		speed (10^6 base per second)		cache miss per base	
Movi	8.5	x4.7	11.29	x 30	1.61	-
SPUMONI	1.8	-	0.38	-	23.30	x 14.47

Table S 3: Related to Figure 1a. Comparing the cache misses between Movi and SPUMONI .

operation	mode	mean	sd	max
fast-forward	Movi-default	0.4189303	3.682711	7694
	Movi-constant	0.3395261	0.913855	9
repositioning	Movi-default	1.039042	2.416862	4345
	Movi-constant	0.4068258	0.4912419	1

Table S 4: Related to Figure 2. Fast-forward and repositioning statistics for the Zymo sample.

sample	# of reads	index	Fulgor (hh:mm:ss)	SPUMONI (hh:mm:ss)	Movi (hh:mm:ss)
pbsim2 simulated	1,188,163	hprc 1	00:17:41	02:43:53	00:06:52
		hprc 94	00:20:38	02:53:23	00:07:28
pbsim2 + gut microbiome	12,204,191	hprc 1	01:46:59	28:06:43	01:01:42
		hprc 94	02:02:17	28:35:48	01:03:06

Table S 5: Related to Section 3.5. Query speed for the human pangenome (HPRC) indexes. One index is built on one haplotype while the other index includes all the 94 haplotypes from the human pangenome. The simulated sample consists of long reads simulated by PBSIM2 [1] from the human genome (CHM13v2 [2]). The second experiment includes both the pbsim2 simulated reads and the real reads from a human gut microbiome sample (`SRR9847854` [3]). The reads are searched in each index for host depletion (removing the human reads).

Tool	Command
SPUMONI	build -i <reference files list> -o <index prefix> -P -n
SPUMONI 2	build -i <reference files list> -o <index prefix> -P
Fulgor	build -l <reference files list> -o <output prefix> -k 31 -m 19 -t 16
minimap2	-x map-ont -d <index file> <reference file>
Bowtie2	–large-index <reference file> <index prefix>
Movi	1) prepare_ref <reference files list> <clean fasta> list
	2) pfp_thresholds -f <clean fasta>
	3) movi-default build default <clean fasta> <index directory>

Table S 6: Related to Section 3. Commands used for building the indexes.

Tool	Command
SPUMONI	run -r <index prefix> -p <reads file> -P -n
SPUMONI 2	run -r <index prefix> -p <reads file> -P
Fulgor	query -i <index file> -q <reads file> -o <output file> -t 1
minimap2	–secondary=no -t 16 -x map-ont <index file> <reads> -o <output>
Movi	query-pf <index directory> <reads file>

Table S 7: Related to Section 3. Commands used for running queries.

Tool	Repository
SPUMONI	`https://github.com/oma219/spumoni` – version 2.0.7
Fulgor	`https://github.com/jermp/fulgor` – version 1.0.0
minimap2	`https://github.com/lh3/minimap2` – version 2.26 (r1175)
Bowtie2	`https://github.com/BenLangmead/bowtie2` – version 2.5.1
pfp_thresholds	`https://github.com/maxrossi91/pfp-thresholds` – version 1.0.0
Movi	`https://github.com/mohsenzakeri/Movi` – version 1.0

Table S 8: Related to Section 3. The tools used in the benchmarks.

Supplementary References

[1] Ono, Y., Asai, K. & Hamada, M. Pbsim2: a simulator for long-read sequencers with a novel generative model of quality scores. *Bioinformatics* 37, 589–595 (2021). https://doi.org/10.1093/bioinformatics/btaa835

[2] Nurk, S. et al. The complete sequence of a human genome. *Science* 376, 44–53 (2022). https://doi.org/10.1126/science.abj6987

[3] Moss, E. L., Maghini, D. G. & Bhatt, A. S. Complete, closed bacterial genomes from microbiomes using nanopore sequencing. *Nature Biotechnology* 38, 701–707 (2020). https://doi.org/10.1038/s41587-020-0422-6
